# Supplementary material for: Relationships between the ground reaction force during initial sprint acceleration and the vertical force–velocity profile
Source: PLoS One. 2025 Jul 15;20(7):e0328225. doi: 10.1371/journal.pone.0328225 (PMC12262837; doi:10.1371/journal.pone.0328225)
Supplement: Attachment — Submitted filename: 31_PONE-D-24-18473_R1_reviewer_revised.pdf [file pone.0328225.s003.pdf]

Title: “Relationships between the ground reaction force during initial sprint acceleration and the vertical force–velocity profile”

The work done by Hirayama and colleagues focuses on the relationship between lower limb mechanical capabilities and the ground reaction forces from the force-velocity profile for the first, fifth and ninth steps during sprint acceleration. The force-profile by squat jumps has been obtained. Although the paper is well-written in standard technical English, in my opinion, there are some parts that should be improved for a better comprehension from the reader, also the discussion should be carefully revised.

1. First of all, the importance of analyzing this relationship should be more stressed by the authors. As it is, it is not clear if its relevance lies in the possibility to improve performance of the athlet or for prevention of injuries or others. Please, try to stress this point in all sections, in particular in the abstract, introduction and conclusion.

2. Methodology, page 7, lines 124-126: Were the participants instructed about the need of the full contact with the platform or not? Subejcts tend to change their natural way of moving if they think about where they have to touch with their feet.

3. Methodology, page 7, line 133: Authors were stated that markers were placed only on the left ilium and on the left foot, in this way they could follow the sagittal movement using only one digital camera, not optoelectronic, right? So, how was the movement of the marker reconstructed if a normal digital camera was used? And why did you need reflective markers if no optical system was employed? Please, clarify this point. Moreover, have you thought about the possibility to employ one inertial measurement unit placed on the wrist, on the sacrum or on the left ilium below the marker to record the acceleration of the subject (see for instance `https://www.scopus.com/record/display.uri?eid=2-s2.0-85201149252&origin=recordpage`, `https://www.scopus.com/record/display.uri?eid=2-s2.0-85088707045&origin=AuthorEval&zone=hIndex-DocumentList`)? Measures from this kind of sensor are reliable and affordable and their use is spread in different activities (`https://europepmc.org/article/med/7723646`, `https://www.scopus.com/record/display.uri?eid=2-s2.0-85201149252&origin=recordpage`, `https://www.scopus.com/record/display.uri?eid=2-s2.0-85088707045&origin=AuthorEval&zone=hIndex-DocumentList`, `https://www.scopus.com/record/display.uri?eid=2-s2.0-85211921394&origin=recordpage`). Authors should properly discuss the possibility to include this kind of sensors for this type of analysis in future studies.

4. Page 8, line 146: I suggest to put the formula of GRF in the equation form with an associated number.

5. Page 8, line 154: “SJ” the title should be in the extended form (i.e. Squat Jump). In case, authors can put the abbreviations in brackets.

6. It is not fully clear to me the rationale behind the necessity to computed the F-V profile with the squat jumps, and which is the correlation with the sprint acceleration. Authors should better clarify the correlation between the SJ, the sprint acceleration and how the F-V profile connects them.

7. Page 9, lines 187-189: Also in this case, one possibility for the future could be the employment of IMU on the sacrum for a rough estimate of the COM displacement (`https://www.scopus.com/record/display.uri?eid=2-s2.0-85088707045&origin=AuthorEval&zone=hIndex-DocumentList`)

8. Page 11, line 206: I suggest to explain better these figures, and how to interpret them.

9. Statistical Analysis, Page 11- beginning of 12: Put reference to Table I at the end of the very first sentence, in this way it is more clear that authors are referring to those metrics. Moreover, in my opinion, it is not correct to state that a correlation values between 0.3 and 0.5 is a moderate correlation, since a value below 0.5 means that there is no correlation at all, but the it is almost completely random, it starts to become a moderate correlation when ρ values are higher than 0.6. However, authors referred to what has been done by Hopkins, but for future studies I suggest to reconsider how the interpretation of the correlation coefficients.

10. Page 13, Figs.4-5: Some plots report the regression line, some others not, is this related to the value of the correlation coefficient? Please, specify. Confidence limits should be indicated in the figures. It seems not so high correlations actually.

11. Page 16, lines 309-311: Are the authors talking about sprint acceleration or SJ. It is not clear in this part, please revise it carefully.

12. Page 16, line 315: Authors stated that reasons of the absence of correlation between V_0 and GRF is unknown, do the authors have any idea about this? Please try to include a possible explanation of this.

13. Future studies section to overcome limitations and to go further with the study should be included at the end of the discussion section.
