# Supplementary material for: Relationships between the ground reaction force during initial sprint acceleration and the vertical force–velocity profile
Source: PLoS One. 2025 Jul 15;20(7):e0328225. doi: 10.1371/journal.pone.0328225 (PMC12262837; doi:10.1371/journal.pone.0328225)
Supplement: Attachment — Submitted filename: Response to Reviewers (1).docx [file pone.0328225.s004.docx]

**Response to the reviewer**

**Reviewer #3**

The work done by Hirayama and colleagues focuses on the relationship between lower limb mechanical capabilities and the ground reaction forces from the force-velocity profile for the first, fifth and ninth steps during sprint acceleration. The force-profile by squat jumps has been obtained. Although the paper is well-written in standard technical English, in my opinion, there are some parts that should be improved for a better comprehension from the reader, also the discussion should be carefully revised.

Response: We are grateful for your detailed and insightful comments, which have contributed significantly to the improvement of our manuscript. We have highlighted our responses to all points in blue. Additionally, the revisions to the manuscript have been emphasized with **bold text and underlines**. We hope our responses and revisions meet your expectations.

1. First of all, the importance of analyzing this relationship should be more stressed by the authors. As it is, it is not clear if its relevance lies in the possibility to improve performance of the athlet or for prevention of injuries or others. Please, try to stress this point in all sections, in particular in the abstract, introduction and conclusion.

Response: The relevance in this study lies in the possibility of improving athletes’ performance. To stress this point, we have revised the manuscript as follows.

Abstract (Lines 39-41):

“Thus, strength training tailored to neuromuscular demands for each step may be effective for **enhancing** sprint acceleration **performance**.”

Introduction (Lines 43-48):

“Sprint acceleration **performance** is essential for field sports athletes. Although the horizontal velocity of the athlete’s center of mass continues to increase from the start of sprint acceleration until approximately 30 m [1], the mean distance of sprint activities is between 10 and 20 m during competitions in many field sports [2]. Hence, to enhance the sprint acceleration **performance** in field sports, the sprint acceleration in a short distance (i.e., initial sprint acceleration) must be improved.”

Discussion (Lines 372-377):

“The present study revealed the phase-specific demands of physical factors for greater GRFs during sprint acceleration, implying that training interventions tailored to different steps are vital for effectively improving the initial sprint acceleration **performance**. The present findings imply that training interventions tailored to steps during sprint acceleration are important to effectively improve sprint acceleration **performance**.”

Conclusion (Lines 395-398):

“These results may help coaches design strength and conditioning programs to improve specific mechanical capabilities that influence the force production in each step during initial sprint acceleration**, thereby effectively enhancing sprint acceleration performance**.”

2. Methodology, page 7, lines 124-126: Were the participants instructed about the need of the full contact with the platform or not? Subejcts tend to change their natural way of moving if they think about where they have to touch with their feet.

Response: In this study, participants were simply instructed to sprint at maximal effort, without being told to aim for full contact with the force plate. As you correctly pointed out, when a single force plate is used to measure ground reaction forces (GRFs) during sprint running, participants may alter their natural running pattern if they are consciously trying to step on the force plate. To avoid this issue, we adjusted the starting position individually for each participant so that they could make natural contact on the force plate without altering their movement. To clarify this point, we have added the following sentence regarding the measurement of 15-m sprint acceleration to the manuscript (Lines 125-127):

“For each step measurement, the starting line was adjusted individually for each participant to ensure that they could naturally contact the force plate without altering their sprint motion.”

3. Methodology, page 7, line 133: Authors were stated that markers were placed only on the left ilium and on the left foot, in this way they could follow the sagittal movement using only one digital camera, not optoelectronic, right? So, how was the movement of the marker reconstructed if a normal digital camera was used? And why did you need reflective markers if no optical system was employed? Please, clarify this point. Moreover, have you thought about the possibility to employ one inertial measurement unit placed on the wrist, on the sacrum or on the left ilium below the marker to record the acceleration of the subject (see for instance https://www.scopus.com/record/display. uri?eid=2-s2.0-85201149252&origin=recordpage, https://www.scopus.com/record/display.uri?eid=2-s2.0-85088707045&origin=AuthorEval& zone=hIndex-DocumentList)? Measures from this kind of sensor are reliable and affordable and their use is spread in different activities (https://europepmc.org/article/ med/7723646, https://www.scopus.com/record/display.uri?eid=2-s2.0-85201149252& origin=recordpage, https://www.scopus.com/record/display.uri?eid=2-s2.0-85088707045& origin=AuthorEval&zone=hIndex-DocumentList, https://www.scopus.com/record/ display.uri?eid=2-s2.0-85211921394&origin=recordpage). Authors should properly discuss the possibility to include this kind of sensors for this type of analysis in future studies.

Response: Thank you for your thoughtful comment and for suggesting relevant literature. One of the purposes of our sprint measurement in this study was to quantify the mean leg extension velocity during the propulsive time (i.e., the phase with a positive horizontal GRF) in sprint acceleration. To achieve this, we placed reflective markers on two anatomical landmarks—the left ilium and the left distal fifth metatarsal— and captured sagittal plane sprint motion using a standard digital video camera. We then defined leg length as the distance between these two points, and calculated the mean leg extension velocity as the change in this distance from the start of the propulsive phase to takeoff, divided by the propulsive time. To ensure accurate manual digitization of anatomical landmarks from video, reflective markers were used to improve their visual clarity. While we did not employ an optoelectronic system, the use of reflective markers in combination with a digital video camera represents a commonly used and traditional approach for obtaining two-dimensional (2D) coordinate data of anatomical landmarks in 2D motion analysis (e.g., <https://pubmed.ncbi.nlm.nih.gov/11404657/>).

As you suggested, inertial measurement units (IMUs) are indeed highly valuable for measuring acceleration, velocity, and estimating joint angles or center-of-mass movement. We fully acknowledge that IMUs are cost-effective and reliable tools, particularly when such parameters are of interest. However, in the present measurement of sprint acceleration, we needed to obtain the 2D coordinate data of the markers to calculate the leg length as defined above. Therefore, rather than using IMUs, which measure acceleration and angular velocity, we considered it more appropriate to use a digital video camera and manual digitization, as this method directly yields the 2D coordinate data necessary for calculating the leg length. In this context, we do not consider the decision not to use IMUs as a methodological limitation of this study, but rather a deliberate methodological choice tailored to the specific parameter we aimed to measure. Therefore, we did not identify the use of IMUs as a direction for future study and we have respectfully decided not to describe IMUs. That said, we sincerely appreciate your suggestion and the references you provided. They reminded us of the practical advantages of IMUs, and we will keep this in mind when conducting more detailed analyses of sprint acceleration mechanics in other studies.

4. Page 8, line 146: I suggest to put the formula of GRF in the equation form with an associated number.

Response: Thank you for your suggestion. We have revised the corresponding part as follows (Lines 147-153):

“The mean angle of the GRF vector during the propulsive phase was calculated as follows:

***θ*_GRF_ = tan^−1^ (GRF_V_/GRF_H_)·180/π**	**(1)**

**where *θ*_GRF_ is the mean angle of the GRF vector and GRF_V_ and GRF_H_ are the mean vertical and horizontal GRFs during the propulsive phase, respectively.**”

In addition, although it has not been pointed out, we have revised the descriptions regarding the calculation of the force-velocity (F-V) profile and the dynamic lower-limb strength to ensure consistent formatting and presentation of equations throughout the manuscript.

Calculation of the F-V profile obtained from squat jumps (Lines 205-217):

“The force and velocity for each loading condition were modeled by least-squares linear regressions to determine the individual F-V profile (Fig 3A):

***F* = *F*_0_ – *αV***	**(2)**

where *F* and *V* represents the mean vertical GRF and leg extension velocity during a SJ at a given load, *F*_0_ represents the theoretical maximum force (force–axis intercept), and *α* is the slope of the linear F-V relationship. Because the F-V relationship was highly linear, the theoretical maximum power (*P*_max_) was determined as**:**

***P*_max_ = *F*_0_·*V*_0_/4**	**(3)**

where *V*_0_ represents the theoretical maximum velocity (velocity–axis intercept) **[9]**.”

Calculation of dynamic lower-limb strength (Lines 231-238):

“As a dynamic lower-limb strength, the mean GRF in the SJ corresponding to the mean leg extension velocity for the first step during sprint acceleration (*F*_1st_) was calculated for each participant using the **linear regression equation of the F-V profile (equation 2)**:

***F*_1st_ = *αV*_1st_ + *F*_0_**	**(4)**

where *α* is the regression coefficient; *V*_1st_, the mean leg extension velocity for the first step during sprint acceleration; and *F*_0_, the theoretical maximum force.”

5. Page 8, line 154: “SJ” the title should be in the extended form (i.e. Squat Jump). In case, authors can put the abbreviations in brackets.

Response: This has been changed in the manuscript．

6. It is not fully clear to me the rationale behind the necessity to computed the F-V profile with the squat jumps, and which is the correlation with the sprint acceleration. Authors should better clarify the correlation between the SJ, the sprint acceleration and how the F-V profile connects them.

Response: As described in the second paragraph of the Introduction, it was assumed that the magnitude of GRFs during sprint acceleration is closely associated with the mechanical capabilities of the lower limbs (i.e., maximal strength or power). However, as outlined in the third and fourth paragraphs, this study aimed not only to examine the relationships with maximal values of the mechanical capabilities, but also to explore the association between GRFs and muscle strength at leg extension velocities that occur during sprint acceleration. Traditionally, the mechanical capabilities of the lower limbs have been assessed under a single condition, such as one-repetition maximum of back squat or unloaded vertical jump. However, the leg extension velocity observed in these tests does not necessarily correspond to that which occurs during sprint acceleration for each individual. Therefore, it was necessary to individually determine a F-V profile based on performances under multiple loading conditions. This approach allowed us to estimate the lower-limb strength at the leg extension velocities observed during sprint acceleration for each individual and to each step (for reference, the figure below reproduces the illustration included in the manuscript showing how these calculations were performed).

[Fig. (A) Typical example of a force–velocity profile obtained from squat jumps. Each closed circle plot represents raw mean force and velocity during a squat jump at a given load. *F*_0_, theoretical maximum force; *P*_max_, theoretical maximum power; *V*_0_, theoretical maximum velocity. (B) Typical example of calculation of dynamic lower-limb strength (*F*_1st_, *F*_5th_, and *F*_9th_) corresponding to the mean leg extension velocities for the first, fifth, and ninth steps (*V*_1st_, *V*_5th_, and *V*_9th_) during sprint acceleration.]

In addition, previous studies that investigated the relationship between sprint acceleration kinetics and lower-limb mechanical capabilities (references 11–14) evaluated these capabilities using F-V profiles derived from SJs. Therefore, in the present study, we adopted the same approach and determined the F-V profiles based on SJs.

Based on the above discussion, we have revised the third paragraph of the Introduction as follows (Lines 73-77):

　　“Thus, a deeper understanding of the contributions of the maximal mechanical capabilities of lower-limb muscles to the GRF during sprint acceleration can be obtained **by using individual F-V profiles, which allows for estimating of potential lower-limb strength at the leg extension velocity observed during sprint acceleration**.”

7. Page 9, lines 187-189: Also in this case, one possibility for the future could be the employment of IMU on the sacrum for a rough estimate of the COM displacement (https:// www.scopus.com/record/display.uri?eid=2-s2.0-85088707045&origin=AuthorEval& zone=hIndex-DocumentList)

Response: As you pointed out, it is certainly possible to estimate COM displacement during the squat jump using an IMU. However, to maintain consistency with the measurement and analysis methods used for sprint acceleration, we chose to calculate COM displacement from marker data in the squat jump as well. As we also mentioned in our response to Comment 3, we do not necessarily regard the absence of IMU as a methodological limitation in the context of this study. For this reason, we have not considered it essential to propose the use of IMU as a direction for future research and we have respectfully chosen not to include a description of IMU-based measurement in the manuscript. We truly appreciate your thoughtful suggestion and hope for your kind understanding.

8. Page 11, line 206: I suggest to explain better these figures, and how to interpret them.

Response: To help readers correctly understand and interpret Fig 3, we have included additional explanations in its caption (Lines 219-228):

“Fig 3. (A) Typical example of a force–velocity **(F-V)** profile obtained from squat jumps. Each closed circle plot represents raw mean force and velocity during a squat jump at a given load. **The F-V relationship for this participant was *F* = *F*_0_ – *αV*, where *F*_0_ (theoretical maximum force) was 38.0 N/kg and *α* was 14.6 Ns/m.** *P*_max_ **(24.8 W/kg)**, theoretical maximum power; *V*_0_ **(2.60 m/s)**, theoretical maximum velocity. (B) Typical example of calculation of dynamic lower-limb strength (*F*_1st_, *F*_5th_, and *F*_9th_) corresponding to the mean leg extension velocities for the first, fifth, and ninth steps (*V*_1st_, *V*_5th_, and *V*_9th_) during sprint acceleration. **The *F*_1st_ (24.4 N/kg) for this participant was calculated by substituting the *V*_1st_ (0.93 m/s) for the F-V relationship. Similarly, the *F*_5th_ (19.7 N/kg) and *F*_9th_ (18.8 N/kg) were obtained using the values of *V*_5th_ (1.26 m/s) and *V*_9th_ (1.31 m/s).**”

9. Statistical Analysis, Page 11 beginning of 12: Put reference to Table I at the end of the very first sentence, in this way it is more clear that authors are referring to those metrics. Moreover, in my opinion, it is not correct to state that a correlation values between 0.3 and 0.5 is a moderate correlation, since a value below 0.5 means that there is no correlation at all, but the it is almost completely random, it starts to become a moderate correlation when ρ values are higher than 0.6. However, authors referred to what has been done by Hopkins, but for future studies I suggest to reconsider how the interpretation of the correlation coefficients.

Response: Thank you for your constructive comment. We have added a reference to Table 1 at the end of the first sentence in the "Statistical analyses" section. The interpretation of correlation coefficients is inherently subjective, and there are no universally accepted absolute standards. In this sense, both our classification of *r* = 0.3–0.5 as “moderate” and your suggestion of a 0.6 threshold may be considered reasonable interpretations. Moreover, there are arguments that the thresholds used to interpret the magnitude of correlation coefficients should be discipline-specific, as the meaning of correlation strength may vary across research fields. For example, Hemphill (*Am Psychol*. 2003) collected correlation coefficients reported in 380 meta-analyses in the field of psychology, and, based on the distribution of these coefficients divided into lower, middle, and upper thirds, proposed the following thresholds: *r* < 0.2 as small, 0.2 ≤ *r* ≤ 0.3 as moderate, and *r* > 0.3 as large. Similarly, Lovakov & Agadullina (*Eur J Soc Psychol*. 2021) analyzed 12,170 correlation coefficients from 134 meta-analyses in social psychology, and proposed thresholds based on quartiles of the distribution: *r* = 0.12, 0.24, and 0.41 for small, medium (moderate), and large, respectively. These studies suggest that the interpretation thresholds for correlation coefficients may vary depending on the field of study. However, to the best of our knowledge, no such investigations have been conducted specifically in the field of sports science. While we understand your suggestion to reconsider how correlation coefficients are interpreted, we believe that using the interpretation proposed by Hopkins et al., which is widely accepted in our field, remains a reasonable and appropriate choice—at least at this time. Nevertheless, we appreciate your comment and will keep this perspective in mind when interpreting the magnitude of correlation coefficients in future studies. We hope our explanation clarifies our position, and we would appreciate your understanding.

[Reference]

Hemphill JF. Interpreting the magnitudes of correlation coefficients. Am Psychol. 2003;58(1):78-9.

Lovakov A., Agadullina E. R. Empirically derived guidelines for effect size interpretation in social psychology. European Journal of Social Psychology. 2021; 51(3):485–504.

10. Page 13, Figs.4-5: Some plots report the regression line, some others not, is this related to the value of the correlation coefficient? Please, specify. Confidence limits should be indicated in the figures. It seems not so high correlations actually.

Response: We have provided responses to each point inline below.

＞Some plots report the regression line, some others not, is this related to the value of the correlation coefficient? Please, specify.

Yes, this is related to the value of the correlation coefficient. We included regression lines only when the correlation was statistically significant; no lines were shown when no significant correlation was found.

＞Confidence limits should be indicated in the figures.

We have added confidence limits in Fig 4 (left) and Fig 5 (right).

　　＞It seems not so high correlations actually.

As you rightly pointed out, the observed correlation coefficients are not necessarily high. Sprint acceleration performance is influenced not only by lower-limb strength but also by various factors such as biomechanics, intermuscular coordination, and muscle-tendon properties. Similarly, GRFs during sprint acceleration are likely affected by multiple factors, making it difficult to interpret them as being predominantly determined by lower-limb strength or power alone. Nevertheless, as described in the manuscript, this study is, to the best of our knowledge, “the first to explore the underlying lower-limb mechanical capabilities associated with GRFs across several steps during initial sprint acceleration” (Lines 293-294). Moreover, the findings may highlight the potential value of designing “training interventions tailored to steps during sprint acceleration” (Lines 375-376). We believe that the findings of this study offer valuable information for readers interested in enhancing sprint acceleration performance.

11. Page 16, lines 309-311: Are the authors talking about sprint acceleration or SJ. It is not clear in this part, please revise it carefully.

Response: To clarify regarding which task (i.e., sprint acceleration or SJ) was being referred to, we have revised the corresponding section as follows (Lines 329-331):

“Furthermore, Nagahara et al. [26] reported that SJ jump height without external load was significantly correlated with acceleration from the sixth to the tenth steps, but not from the first **to** fifth steps **during 60-m sprint running**.”

12. Page 16, line 315: Authors stated that reasons of the absence of correlation between V0 and GRF is unknown, do the authors have any idea about this? Please try to include a possible explanation of this.

Response: We have revised the relevant part to include a possible explanation for the lack of significant correlations between GRFs during sprint acceleration and *V*_0_ (Lines 334-342):

“In contrast to *P*_max_, no correlation was observed between *V*_0_ and the horizontal and resultant GRFs for all steps. **Morales-Artacho et al. [27] reported that isometric knee extensor torque and the rate of torque development were strongly related to *F*_0_ and *P*_max_ in the F-V profile obtained from countermovement jump, while there were no associations with *V*_0_. Although the jump types used in Morales-Artacho et al. [27] and the current study differed, it is possible that *V*_0_ in vertical jump may not adequately reflect intrinsic force-generating capabilities of the lower-limb, which may explain the lack of significant correlations with GRFs during sprint acceleration.** Our results suggest that the ability to produce larger force and power is more important than the ability to produce higher velocity in the initial sprint acceleration.”

13. Future studies section to overcome limitations and to go further with the study should be included at the end of the discussion section.

Response: We have added future study directions at the end of the discussion section as follows (Lines 383-389):

“**This study has some limitations. First, the study participants were only male baseball players. It has been suggested that several differences in sprint acceleration kinetics exist between different cohorts, such as those in sport [33], sex [34], and age [35]. Thus, the results might differ if athletes from other sports, female athletes, or youth and master athletes were employed, which warrants further investigation. Second, because this is a cross-sectional study, causal relationships remain unclear. Thus, further prospective research is needed to determine whether training-induced changes in the F-V profile can influence GRFs during initial sprint acceleration.**”

As shown above, we have included sentences on future study directions in conjunction with the limitations of this study. We chose this approach because we believe that limitations and future directions are conceptually linked and are best discussed together. We hope this revision meets your expectations.
